# Formulation Development of Mirtazapine Liquisolid Compacts: Optimization Using Central Composite Design

**DOI:** 10.3390/molecules27134005

**Published:** 2022-06-22

**Authors:** Faiza Naureen, Yasar Shah, Sayyed Ibrahim Shah, Muhammad Abbas, Inayat Ur Rehman, Salar Muhammad, Hamdullah, Khang Wen Goh, Fazli Khuda, Amjad Khan, Siok Yee Chan, Mehwish Mushtaq, Long Chiau Ming

**Affiliations:** 1 Department of Pharmacy, Abdul Wali Khan University Mardan, Mardan 23200, Pakistan; faizanaureen@hotmail.com (F.N.); muhammadabbas@awkum.edu.pk (M.A.); inayat.rehman@awkum.edu.pk (I.U.R.); salar_pharmacist@awkum.edu.pk (S.M.); hamdullah@awkum.edu.pk (H.); 2 Faculty of Data Science and Information Technology, INTI International University, Nilai 71800, Malaysia; khangwen.goh@newinti.edu.my; 3 Department of Pharmacy, University of Peshawar, Peshawar 25000, Pakistan; fazlikhuda@uop.edu.pk (F.K.); mahmushtaq@gmail.com (M.M.); 4 Department of Pharmacy, Kohat University of Science & Technology, Kohat 26010, Pakistan; amjadph@gmail.com; 5 School of Pharmaceutical Sciences, Universiti Sains Malaysia, Minden 11800, Malaysia; sychan@usm.my; 6 PAP Rashidah Sa’adatul Bolkiah Institute of Health Sciences, Universiti Brunei Darussalam, Gadong BE1410, Brunei

**Keywords:** mirtazapine, green products, sustainable manufacturing, in vitro characterization, mental disease, dissolution enhancement, poorly water-soluble drugs

## Abstract

Mirtazapine is a tetracyclic anti-depressant with poor water solubility. The aim of this study was to improve the dissolution rate of mirtazapine by delivering the drug as a liquisolid compact. Central composite design (CCD) was employed for the preparation of mirtazapine liquisolid compacts. In this, the impacts of two independent factors, i.e., excipient ratio (carrier:coating) and different drug concentration on the response of liquisolid system were optimized. Liquisolid compacts were prepared using propylene glycol as a solvent, microcrystalline cellulose as a carrier, and silicon dioxide (Aerosil) as the coating material. The crystallinity of the formulated drug and the interactions between the excipients were examined using X-ray powder diffraction (XRD) and Fourier-transform infrared spectroscopy (FTIR), respectively. The dissolution study for the liquisolid compact was carried out as per FDA guidelines. The results showed loss of crystallinity of the mirtazapine in the formulation and was completely solubilized in non-volatile solvent and equally dispersed throughout the powder system. Moreover, drug dissolution was found to be higher in liquisolid compacts than the direct compressed conventional tablets (of mirtazapine). The liquisolid technique appears to be a promising approach for improving the dissolution of poorly soluble drugs like mirtazapine.

## 1. Introduction

The oral route is the most preferred means of the drug administration due to the higher patience compliance and low cost of production [1,2]. The drug must be in solution form for absorption through gastrointestinal tract (GIT) when given orally [3]. In case of poorly soluble drugs, dissolution is the rate-limiting step in absorption process [4]. Generally, compounds with aqueous solubility lower than 100 μg/mL show dissolution-limited absorption from the GIT of humans [5,6].

The concept of liquisolid tablets was developed initially from powdered solution technology that can be used to formulate liquid medication. A liquisolid system is defined as dry, non-adherent, free-flowing and compressible powder mixtures converted from liquid drugs, drug suspensions or drug solutions in nonvolatile solvents with selected carriers and coating materials [7,8,9]. In this technique, the drug is dissolved in a non-volatile solvent and converted into a dry, free flowing, and compressible solid using carrier and coating materials. Moreover, since non-volatile solvents are used to prepare the drug solution/suspension, the liquid is not evaporated and the drug is carried in a liquid system and is dispersed throughout the final product [10,11]. Liquisolid technique, compared to other conventional oral formulations, is of lower cost with more sustainable manufacturing processing [12].

Mirtazapine, [±]-2-methyl-1,2,3,4,10,14b-hexahydropyrazino [2,1-a]pyrido [2,3-e] benzazepine, is a water insoluble tetracyclic antidepressant with a 20–40 h half-life [13,14]. Various studies to improve the dissolution profile of the drug, i.e., mirtazapine, by preparation of orally disintegrating tablets (by using polymers like Eudagrid at a concentration of 6% *w/w*) or using the sublimation technique has been reported, with results showing enhanced dissolution profile [15,16,17]. Although these approaches increased the release of the drug from solid dosage form, it was not more than 70%. For this purpose, a method was required that can possibly further increase the dissolution of the drug [15]. Thus, the rationale behind preparation of liquisolid compacts was to further increase the dissolution profile of mirtazapine.

One of the crucial steps in formulation development is the design and optimization of the formulation. For this purpose, various approaches can be used. The conventional approach is to change a single factor or variable while keeping the other independent factors constant to observe the effect of composition. However, this approach requires many experiments, and the interaction between factors is difficult to study; the results of the experiments could also be misinterpreted [18]. To overcome this problem, the application of mathematical models, e.g., design of experiment methods, such as central composite design (CCD) during the design and development process could determine the interactive effect of different variables that can influence the results/quality of the formulation. That mathematical model helped to calculate the amount of excipient with appropriate properties. Moreover, CCD has been successfully used in several studies for the development and optimization of formulations, as data obtained using CCD showed reliable predictions [19,20]. Two independent factors (excipients ratio and drug concentration in liquid medication) with two levels (low and high) according to central composite design (CCD) were employed for designing the liquisolid formulations of mirtazapine. CCD consisted of groups of experiments based on factorial, axial, and central points, according to desired properties of the design. It allowed to evaluate the effects of two independent factors (ratio of excipient and the concentration of the drug in non-volatile solvent) for three dependent variables (responses), i.e., the angle of repose, disintegrating time, and the drug release (dissolution) from tablets in 30 min. Optimum results were obtained using CCD for the liquisolid technique.

The objective of this study was to analyze the use of the liquisolid technique in improving the solubility and dissolution profile of a drug by designing it as liquisolid compacts, and then compressing it into tablets via direct compression.

## 2. Methods

### 2.1. Saturated Solubility

Saturation solubility studies for mirtazapine were carried out in triplicate in five different non-volatile solvents, i.e., polyethylene glycol 400 (PEG-400), glycerin, propylene glycol, Tween-80, and Span-80. The highest amount of the drug (mg) solubilization (mL^−1^) was noted in the respective solvent.

### 2.2. Preparation of Liquisolid Compacts

The desired quantity of the drug was accurately weighed and dissolved in the liquid vehicle (non-volatile solvent). The non-volatile solvent was selected based on saturated solubility. That solution was then sonicated for 15 min until a homogeneous drug solution was obtained. The solution was heated slightly to obtain a clear drug solution, which was then followed again by sonication. After that, the calculated weights (W) of the resulting liquid medications, equivalent to the doses of the drug, i.e., 15 mg (3.9 g), 30 mg (7.9 g) and 45 mg (12.6 g), were incorporated into the calculated quantities of the carrier material, i.e., Avicel PH 102, and was mixed thoroughly. A wet mixture was formed, which was then blended with the calculated amount of the coating material, i.e., Aerosil (silicon dioxide), using a standard mixing process to form a simple admixture.

Different liquisolid formulations were designed based on two factors, i.e., concentration of the drug in liquid vehicle (non-volatile solvent) and carrier:coating ratios. Different liquid load factors (Lf) were employed for these different formulations. Finally, 5% *w*/*w* of sodium starch glycolate was mixed with the above mixtures for 10 min.

The final blend of liquisolid powder system was compressed into tablets of desired weight (15 mg, 30 mg and 45 mg strength), each using a tablet compression machine (ZP 19 tablet rotary press machine (China). The tablets had round-shaped punches and 8 mm sized dies.

### 2.3. Preparation of Direct Compressed Tablets

Directly compressed conventional tablets of mirtazapine were also prepared for comparison with liquisolid compacts. These tablets were prepared by directly compressing the powder mixture of Mirtazapine with Avicel PH 102 Aerosil, polyvinyl-pyrrolidone and sodium starch glycolate. The same round-shaped punches with a die size of 8 mm were used.

### 2.4. Experimental Design for Liquisolid Compacts Formulations

Central composite design (CCD) was employed for the preparation of mirtazapine liquisolid compacts. In the design, the impacts of two independent factors, i.e., excipient ratio (carrier:coating) and different drug concentrations, on the responses of the liquisolid system were optimized.

The complete design was executed in a random order and comprised 11 combinations with three replicates at a central point, and two experiments on an axis associated with each factor at the level of xi = ±α. The levels for CCD and their coded values are given in Table 1. Experimental data were analyzed using a multiple regression equation to fit a second-order polynomial model [21,22].

The model (Equation (1)) used is given below:

(1) $$Y=\beta_{0}+\overset{n}{\underset{i=0}{\sum }}\beta_{i}X_{i}+\overset{n}{\underset{i=0}{\sum }}\beta_{ii}X_{i}^{2}+\overset{n}{\underset{i\neq i=1}{\sum }}\beta_{ij}X_{i}X_{j}$$

where *Y* is the predicted response, $\beta_{0}$ is the intercept, *n* is the number of factors analyzed, and $\beta_{i}$, $\beta_{ii}$ and $\beta_{ij}$ are the linear (main effect), quadratic, and interactive model coefficients, respectively. Accordingly, *X_i_* and *X_j_* indicate the levels of independent parameters.

### 2.5. Mathematical Model for Designing Mirtazapine Liquisolid Formulation

According to the mathematical model, the required ingredient quantities and the flowable liquid-retention potentials (F-values) of the powder excipients were calculated [23,24]. These values were used for obtaining desirable liquisolid formulations. The flowable liquid-retention potentials for Avicel PH 102 and Aerosil were 0.294 and 8.965, respectively [24].

The liquid load factor was calculated from the flowable liquid-retention potential in accordance with Equation (2), using an R value (excipient ratio).

Lf = F1 + F2 (1/R)(2)

where Lf = liquid load factor.

F1: Flowable liquid retention potential of carrier;

F2: Flowable liquid retention potential of coating material;

The most suitable quantities of carrier (Q) were calculated using Equation (3):

Lf = W/Q(3)

W= weight of liquid medication;

Q = amount of carrier material.

The optimum quantities of carrier (Q) and coating material (q) were obtained with Equation (4):

R= Q/q(4)

q = amount of coating material.

The compositions of different liquisolid systems, based on the mathematical model and central composite design, are given in Table 2.

### 2.6. Formulation and Characterisation of Mirtazapine Liquisolid Compacts

#### 2.6.1. Flow Properties of Liquisolid Systems

The flow properties of liquisolid systems were assessed using the angle of repose, Carr’s index, and the Hausner ratio [25].

The angle of repose was estimated using a fixed funnel. The powder was poured into the funnel and a free-standing cone was allowed to form. The angle of the cone was then calculated. The Hausner ratio was estimated from the bulk density of the powder and Carr’s density was calculated from the tap density.

#### 2.6.2. Differential Scanning Calorimetry (DSC)

DSC was conducted to assess the heat properties of the drug when converting to a new formulation (liquisolid) (universal V2.4F, Thermal Analysis (TA) Instruments, Delaware, USA). 5 mg of sample was placed in hermatically-sealed aluminum pan. The rate of heat transfer was kept at 10 °C/min. The range of temperature in DSC was 80 °C to 330 °C and a nitrogen atmosphere was also maintained.

#### 2.6.3. X-rays Powder Diffractometry (XRD)

To determine the crystalline state of drug in liquisolid compacts, an X-ray diffraction study was carried out. For this purpose, a Philips analytical X-ray diffractometer (Model: PW3711, The Netherlands) was used. For this study, the voltage rate was selected at 40 kV. The scanning angle ranged from 5° to 70°, and the counting rate was chosen to be 0.4 s/step.

#### 2.6.4. Fourier-Transform Infrared Spectroscopy (FTIR)

FTIR is a standard method for analysis for pharmaceutical products to identify any chemical interactions between the drug and excipients in the formulation. FTIR spectra of conventional tablets and liquisolid compacts (that had highest dissolution) were recorded on a Shimadzu FTIR spectrophotometer (Shimadzu Corporation, Kyoto, Japan).

#### 2.6.5. Scanning Electron Microscopy (SEM)

Morphological estimations of conventional tablet of mirtazapine and liquisolid compacts were conducted on a scanning electron microscope (JEOL, Tokyo, Japan).

#### 2.6.6. Hardness of Liquisolid Compacts

Ten tablets from each batch of liquisolid compacts were randomly selected and analyzed in a hardness test. The hardness of the liquisolid compacts was determined using a Monsanto hardness tester.

#### 2.6.7. Friability Test

A single drum Roche friabilator (Faisal Engineering, Lahore, Pakistan) was used to assess the friability of liquisolid compacts according to the pharmacopeia. Liquisolid compacts were randomly selected and weighed. The liquisolid compacts were charged into the friabilator drum and rotated for 4 min at 25 rpm. After 100 revolutions, the liquisolid compacts were unloaded, de-dusted, and weighed again.

#### 2.6.8. Weight Variation Test

Liquisolid tablets were evaluated in a weight variation test, according to an official method [26]. Liquisolid compacts were selected randomly (*n* = 20), weighed separately, and then their average weights determined.

#### 2.6.9. Drug Content Uniformity

Ten tablets were randomly selected from each formulation. They were crushed and powder was transferred to a 100 mL volumetric flask containing 40 mL of methanol. The flask was shaken to dissolve the drug and the volume was adjusted with methanol to obtain a stock solution. Further suitable dilutions were performed. The absorbance of the solution was recorded at a lambda max of 292 nm on a UV spectrophotometer (Shimadzu, UV mini-4201, Japan).

#### 2.6.10. Disintegration Time

The disintegration time of the liquisolid compacts was determined according to USP [27], using a USP tablet disintegration testing apparatus (Pharma Test, Hamburg, Germany). The disintegration medium was distilled water at a temperature of 37 ± 2 °C. Six liquisolid tablets were randomly selected from each batch and the disintegrating time was determined.

#### 2.6.11. In-Vitro Drug Release

For estimating the in vitro drug release, a USP II dissolution apparatus (paddle type) was used. All liquisolid formulations were subjected to dissolution studies under the dissolution conditions, as prescribed by the US FDA. The dissolution rate was analyzed under the conditions of 0.1 N HCl (pH 1.2) and was maintained at 37 ± 2 °C.

The rotation speed of the paddle was maintained a 50 rpm. Samples were taken and filtered at different time intervals (5, 10, 15, 20, 30, 45, 60 min). The amount of drug released was analyzed by measuring the UV absorbance of each sample at 292 nm using a UV-visible spectrophotometer (Shimadzu Corporation, Japan).

## 3. Results and Discussion

### 3.1. Saturated Solubility Study

The saturation solubility of mirtazapine was checked in different non-volatile solvents and solvent systems (mixture of solvents). Propylene glycol was selected for mirtazapine solubility because it solubilized 600 ± 35 mg/mL of the drug. Other non-volatile solvents showed little solubility compared to propylene glycol. The order of solubility was glycerin (100 ± 9 mg/mL) < Tween-80 (80 ± 23 mg/mL) < polyethylene glycol (250 ± 11 mg/mL) < propylene glycol (600 ± 35 mg/mL)

### 3.2. Flow Properties of Mirtazapine Liquisolid Formulation

The flow property of the liquisolid system was also assessed. For that purpose, angle of repose, compression index, and Hausner’s ratio were determined.

The angle of repose was calculated with a fixed funnel and the free-standing cone method. The angle of repose was found in the range of 27 to 36, which indicated an acceptable flow property and was further supported by lower values of Carr index (also Carr’s index or Carr’s compressibility index), as presented in Table 3.

Hausner’s ratio and Carr’s index were also calculated from the bulk density and the tapped density of the powder mixtures. The final predictive equation for the angle of repose was obtained using Design Expert Software, version 13, as shown in Equation (5):

Y1 = + 5.35+ 0.1469A + 0.1956B−0.0060A + 0.1882A^2^ + 0.1736B^2^ (5)

The optimal level effects of the two independent variables on angle of repose were described with three-dimension response surface contour plots (surface response graphs). These showed that two independent variables showed effects on the angle of repose.

As the drug concentration in the liquid increased, the flow property of the powder decreased. For obtaining a good flow property of the powder, increasing the excipient ratio of the liquisolid formulation was needed.

Majority of the formulations showed good flow properties and less compressibility. Values obtained for the Hausner and Carr indices were acceptable. These ranged from 1.17 to 1.35 for the Hausner’s ratio and 14.9 to 26.0 for the Carr’s index.

**Figure 1:**
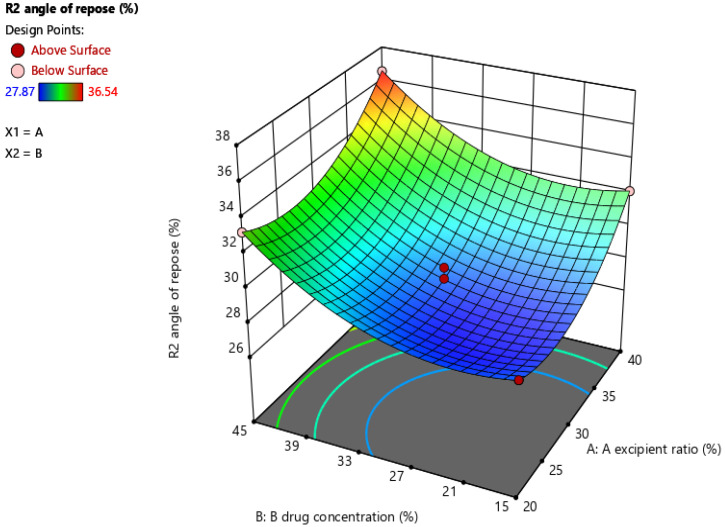
Surface response graph of the angle of repose.

The surface response graph of the angle of repose in Figure 1 shows acceptable flow properties for all liquisolid formulations. It was found that, if the excipient ratio increased and the drug concentration in the liquid medication decreased, then the formulation could show good flow properties.

The significance levels of the two independent factors were found using the central composite design. Analysis of multiple regression and coefficients of the model were applied for determining the significance of the levels of the two factors. Significance was found among the linear, interactive, and quadratic effects of two independent variables (*p* < 0.05).

### 3.3. Hardness of the Tablets

The hardness of all compacts was found to be in the range of 2.67 ± 0.16 to 4.34 ± 0.10 kg/cm^2^. The data are shown in Table 4.

The hardness of the tablets were observed against the added amount of Avicel. It was found that hardness of the tablets increased by increasing the amount of Avicel. Adjusting the quantity of carrier and coating materials increased the hardness of the tablets [28].

When the ratio (R) of the carrier and coating material declined, the hardness of the tablets also decreased. It was clear that, if less quantity of Avicel was added to the formulation, then the desired compressibility could not be obtained with Aerosil alone.

A desired strength and cohesiveness for the compacts is possible if the amount of Avicel is added according to the adjusted ratio of excipients based on central composite design. Increasing the amount of Avicel provides a high bonding power between the hydrogen groups of the cellulose material.

### 3.4. Tablet Dimension

The thickness of the liquisolid compacts ranged from 2.03 to 4.45 mm and the diameter of all liquisolid compacts was found to be in the range of 9.00 to 11.3 mm.

### 3.5. Weight Variation Test

All liquisolid compacts of mirtazapine were evaluated using a weight variation test. According to the USP specification, the weights of all tablets were in an acceptable range [27]. A minor variation showed that all formulations were mixed properly and were compressed effectively. The variation is expressed as mean ± SD in Table 4.

### 3.6. Friability

All liquisolid compacts were evaluated using a friability test. All liquisolid compacts showed adequate friability. None of the tested formulation showed loss in tablet weights greater than 1%. The results are expressed in Table 4.

### 3.7. Content Uniformity Test

All formulations were observed in a content uniformity test. Randomly selected tablets from all formulations were tested for uniformity of contents. Tablets showed uniformity regarding all contents, according USP specifications (85 to 115%). Content uniformity exceeded 99%. The highest level of content uniformity showed that the excipients and drug in all formulations were mixed properly and effectively. Content uniformity is shown in Table 4, along with ± SD.

### 3.8. Disintegrating Time of Liquisolid Compacts

The disintegration test was performed for all liquisolid systems, indicating that all the liquisolid tablet were disintegrated within 15 min. Disintegration of tablets was according to USP specifications for uncoated tablets, given Table 4 [27].

**Figure 2:**
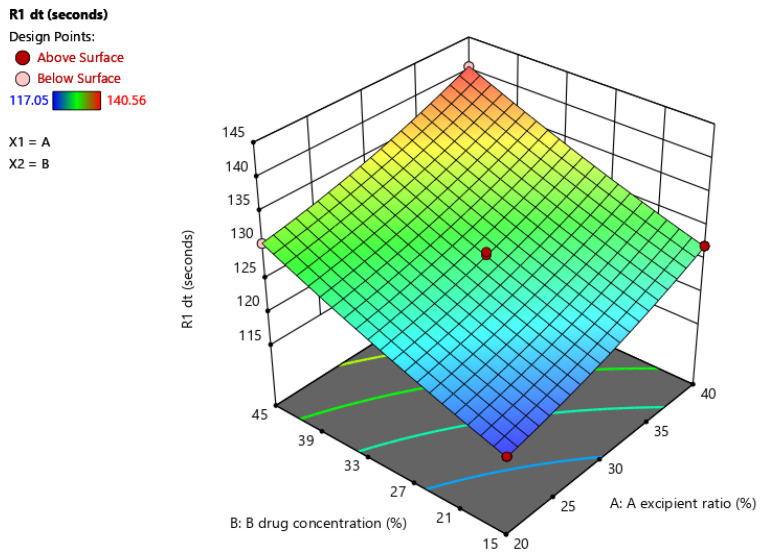
Surface response graph of disintegration time of liquisolid compacts.

The surface response graph in Figure 2 of the disintegration time, as per central composite design (CCD), revealed that, as the excipient ratio (R) and the drug concentration in the liquid increased, the disintegrating time also increased.

Regression values (R^2^ (0.9982)) for the disintegration time were determined using Design Expert Software, version 13, and is presented in Table 4. Independent variables for the angle of repose were quadratic and showed a good regression coefficient, R^2^ (0.9877).

According to statistical analyses of the data, the disintegration time of all formulations was significantly affected by two independent variables. Disintegration time was affected significantly by the linear, quadratic, and interactive variables that were used for designing liquisolid formulation.

The predictive equation for disintegrating time involving significance terms is given in Equation (6):

Y2 = + 11.28 + 0.2230A + 0.2990B−0.0077AB + 0.0438A^2^ + 0.0218B^2^ (6)

Microcrystalline cellulose (Avicel 102) and sodium starch glycolate both worked to accelerate the disintegration of liquisolid compacts and further improved the dissolution of drug.

### 3.9. Ex Vivo Drug Release (Dissolution within 30 min)

**Figure 3:**
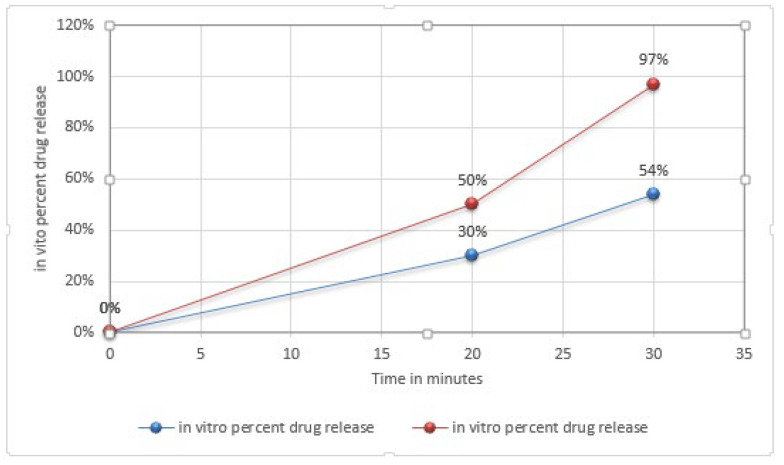
Comparison graph for direct compressed tablets and liquisolid compact. Red line represents percent drug release of conventional tablet, blue line represents percent drug release of liquisolid compact.

Liquisolid compacts showed good dissolution within 30 min. Enhanced dissolution (up to 97%) was found in LS 2 and LS 7, as presented in Table 4. This could increase with a possibility of up to 100% in 45 min. All others formulation also showed a high rate of dissolution (more than 80%) in 30 min. The conventional tablets showed 54% dissolution within 30 min. A comparison graph of the dissolution of both conventional tablets and liquisolid tablets is shown in Figure 3.

High dissolution of liquisolid compacts was due to the solution of the drug, which was equally dispersed in the excipients. Excipients provided maximum wettability and a high surface area for the mirtazapine that was completely dissolved in non-volatile liquid. Subsequently, the drug was released quickly in the dissolution medium [29].

Use of non-volatile solvent (PG) also facilitated the wettability of the drug particles. This mechanism was used by reducing the surface tension between the dissolution medium and tablet.

The linear, quadratic, and interactive effects, as per CCD, indicated that two independent variables (excipient ratio and drug concentration) had an impact on the release of the drug from the solid dosage form. Ex vivo dissolution of the drug was significantly (*p* = 0.05) affected by the two variables; thus, changing the amounts of the two variables, i.e., excipient ratio and drug concentration, caused increases and decreases in drug release in 30 min from the liquisolid compacts.

The linear, quadratic, and interactive effects of the two independent variables (excipient ratio and drug concentration) on drug release were determined with CCD by using Design Expert Software, version 13. All effects of the variables were found to be significant (*p* < 0.05). This means that changing the number of variables caused an increase and decrease in drug release in 30 min from the liquisolid compacts.

Independent variables had a quadratic effect on drug release and showed a good regression coefficient (R^2^ = 0.9951), as shown in Table 5.

The predictive equation for in vitro drug release involving all significance terms is given in Equation (7):

Y3 = + 9.66 + 0.0700A−0.1246B + 0.0131AB + 0.0078A^2^ + 0.0012B^2.^ (7)

**Figure 4:**
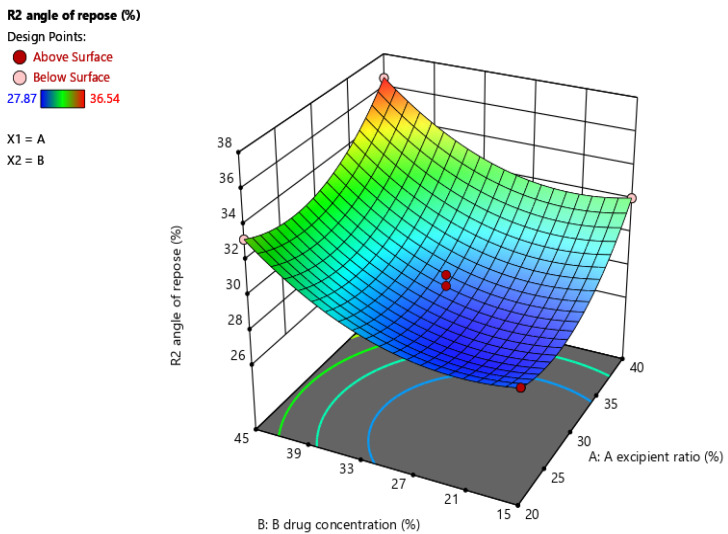
Surface response graph of in vitro dissolution of liquisolid compacts.

A surface response graph for drug released within 30 min is shown in Figure 4.

### 3.10. X-ray Diffractrometry

**Figure 5:**
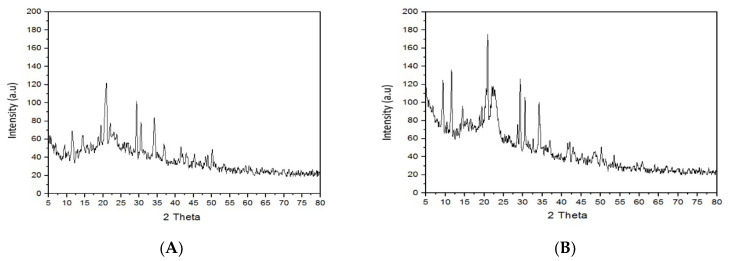
X-ray diffractrogram for pure drug (**A**) and for liquisolid compact (**B**).

X-ray diffractometry was conducted for the pure drug, mirtazapine, and for liquisolid compacts of mirtazapine. The diffractogram in Figure 5 show some peaks that appeared at 9.54, 14.66, 20.24, 21.18, 22.38, 29.0, 30.52, 36.72, etc. The peaks supported the crystalline nature of the drug.

The diffraction marks of the liquisolid compact showed only one high diffraction peak of 20.7. That peak appeared to be due to the crystal state of Avicel PH 102, which was present in the liquisolid compacts [28].

The X-ray diffractogram of liquisolid compact showed an absence of any specific peak. This confirmed the complete conversion of the drug from its crystal sate to an amorphous state. The drug was completely solubilized in liquid in the liquisolid compact, which caused no productive reflection in the diffractogram.

### 3.11. FTIR Spectrum of Liquisolid Compact

**Figure 6:**
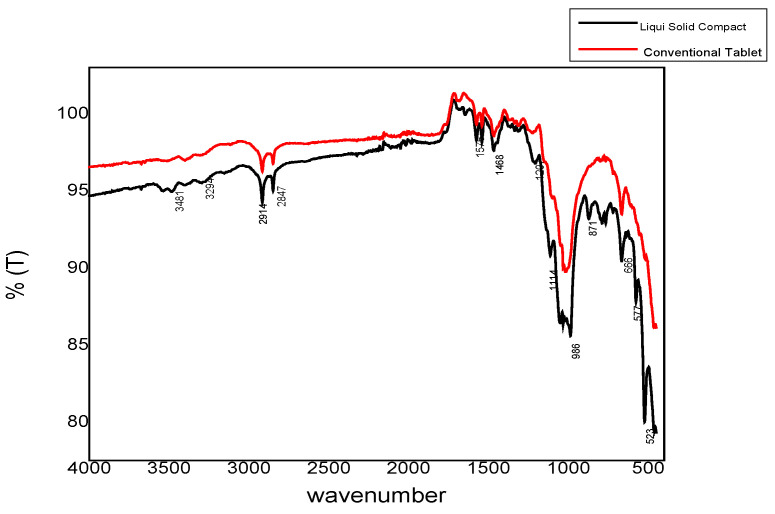
FTIR spectrum for both liquisolid and conventional tablets.

FTIR spectroscopy was done for the conventional tablets and the liquisolid compacts. IR spectra were similar for both tablets. No differences were found in FTIR spectra. Some specific peaks in both spectra were related to ether and carbonyl stretching. The FTIR spectrum is shown in Figure 6. The lack of difference between the two spectra indicated that chemical reactions among the drug and excipients in the liquisolid formulation were not present.

### 3.12. Differential Scanning Calorimetry (DSC)

The DSC graph of mirtazapine depicted a sharp heat-absorbing peak at 117 °C. That peak was related to the melting of the drug and decomposition of the drug. A sharp endothermic peak at 117 °C signified mirtazapine in its pure crystalline state.

**Figure 7:**
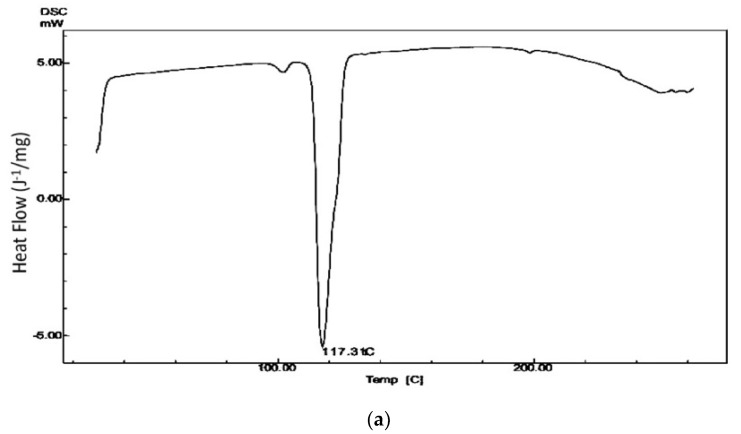

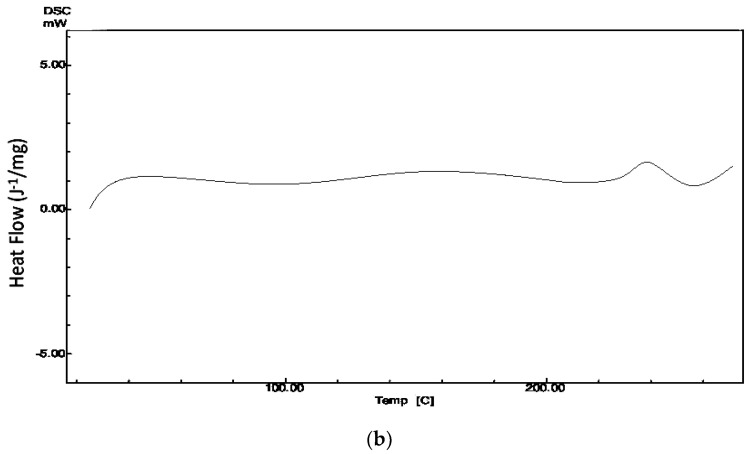
DSC thermograph of pure drug (**a**) and of liquisolid compacts (**b**).

The thermogram of the liquisolid compact in Figure 7 shows a complete disappearance of a peculiar peak. The absence of the distinctive peak clarified that the drug was in a solution form in the liquisolid system and was dispersed molecularly within the liquisolid compacts [30].

### 3.13. Scanning Electron Microscopy (SEM) for Liquisolid Compacts

**Figure 8:**
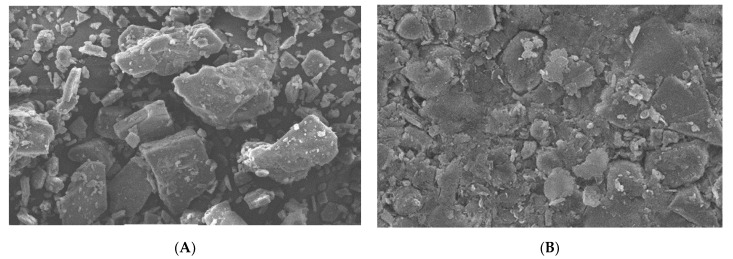
Scanning electron photomicrograph for pure drug (**A**) and for liquisolid compact (**B**).

SEM was conducted for the solid drug, i.e., mirtazapine and for the liquisolid compact. The SEM results proved that the drug had a clearly crystalline nature. It is shown in Figure 8A. The photomicrographs of the liquisolid compact showed a complete absence of crystalline structure of the drug. It can be seen in Figure 8B that the results supported the XRD and DSC outcomes.

The SEM results indicated that the drug was completely dissolved in the liquisolid system.

### 3.14. Verification of Predicted Model

Central composite design suggested some estimated levels for obtaining optimum results for three responses for all liquisolid formulation (Table 6).

Some predicted ratio of excipients and drug concentration in liquisolid formulation was also suggested by the design expert software in order to determine a formulation that can give good results. According to the predicted model, three formulations were prepared, to acquire enhanced dissolution in 30 min, minimum disintegration time, and an excellent angle of repose.

The predicted model was used according to suggested ratio given by the software and experiments were performed. The experiments verified the predicted model. The suggested ratio of the two variables (excipient and drug conc.) were applied for preparation of liquisolid compacts. The same results were obtained, as suggested by the predicted model.

The same results of the experiment confirmed the RSM for the liquisolid preparation. The results obtained from the experiments confirmed that there was no significant difference between the predicted and experiment results. In short, this model can be used to optimize the excipient and drug conc. ratios to prepare liquisolid compacts of mirtazapine.

## 4. Conclusions

The liquisolid technique has been tested in this study to increase the dissolution of mirtazapine (a water insoluble drug). For the preparation of liquisolid compacts, various parameters are important, i.e., excipient ratio, concentration of drug, etc. Conventional mean of optimizing these parameters include performing tedious experiments that take more time and resources. CCD was used to optimize the excipient ratio and concentration of the drug, which led to the preparation of a liquisolid compact formulation of mirtazapine with a dissolution enhancement of up to 97%, as compared to conventional compressed tablets; thus, proving the utility of the liquisolid technique in formulation sciences.

## Figures and Tables

**Table 1:** Levels of independent variables of central composite experimental design.

**Batch**	**Actual Values**			**Coded Values**	
	X1		X2	X1	X2
LS1	30		30	0	0
LS2	40		15	1	−1
LS3	15.857		30	−1.4142	0
LS4	20		15	-	−1
LS5	30		30	0	0
LS6	40		45	1	1
LS7	30		8.786	0	−1.4142
LS8	20		45	−1	1
LS9	30		51.213	0	1.4142
LS10	30		30	0	0
LS11	44.142		30	1.4142	0
Independent variables for liquisolid system					
Independent Variables	Levels				
	−X (−1.414)	Low (−1)	Center (0)	High (+1)	+X (−1.414)
Excipient ratio R% X1 (carr:coat)	15.85	20	30	40	44.14
Drug conc. in liquid X2	8.78	15	30	45	51.21

**Table 2:** Compositions of different liquisolid compacts of mirtazapine according to the mathematical model and CCD.

Liquisolid Systems	Excipient Ratio (R)	% Drug Concentration Cd (% *w*/*w*)	Load Factor (Lf)	Avicel in mg (Q)	Aerosil in mg (q)	SSG in mg	Unit Dose Weight in mg
LS1	30	30	0.272	300.20	10.00	69.30	444.65
LS2	40	15	0.169	240.32	6.00	55.13	356.56
LS3	15.86	30	0.348	240.50	15.20	61.41	387.11
LS4	20	15	0.207	200.31	10.01	47.12	301.64
LS5	30	30	0.452	205.21	6.70	52.24	330.33
LS6	40	45	0.406	320.00	8.01	75.43	486.90
LS7	30	8.78	0.100	223.5	7.40	52.30	331.61
LS8	20	45	0.475	280.10	14.01	68.33	446.42
LS9	30	51.2	0.424	350.62	11.51	82.42	536.14
LS10	30	30	0.389	210.11	7.00	54.33	341.72
LS11	44.14	30	0.237	340.06	7.75	75.01	491.08

**Table 3:** Angle of repose, Carr’s index, Hausner’s ratio of all liquisolid systems.

Liquisolid Systems	Bulk Density (g)	Tapped Density (g)	Angle of Repose	Hausner’s Ratio	Carr’s Index %
LS1	0.46	0.54	28.67 ± 0.01	1.17 ± 0.017	14.9 ± 0.08
LS2	0.41	0.51	32.13 ± 0.40	1.24 ± 0.081	19.7 ± 0.22
LS3	0.39	0.48	30.44 ± 0.34	1.23 ± 0.037	18.8 ± 0.37
LS4	0.35	0.41	28.79 ± 0.05	1.17 ± 0.061	14.7 ± 0.29
LS5	0.37	0.45	29.34 ± 0.23	1.21 ± 0.016	17.8 ±0.09
LS6	0.34	0.45	36.54 ± 0.21	1.32 ± 0.027	24.5 ± 0.28
LS7	0.28	0.34	29.37 ± 0.18	1.21 ± 0.024	17.9 ± 0.41
LS8	0.26	0.34	33.25 ± 0.17	1.30 ± 0.011	23.6 ± 0.08
LS9	0.37	0.49	35.72 ± 0.16	1.32 ± 0.017	24.5 ± 0.24
LS10	0.44	0.52	27.87 ± 0.09	1.18 ± 0.014	15.4 ± 0.22
LS11	0.34	0.46	35.25 ± 0.08	1.35 ± 0.012	26.0 ± 0.29

All values are expressed as mean ± SD (*n* = 3).

**Table 4:** Evaluation of liquisolid compacts of mirtazapine.

Formulation Number	Hardness (kg/cm)^2^	Weight Variation (mg)	Friability (%)	Disintegration Time (s)	% Drug Content	% Drug Release in 30 min
Conventional/control	2.69 ± 0.18	200.2± 0.143	0.45	132.22 ± 0.123	92.8 ± 0.20	54.86 ± 0.335
LS1	3.32 ± 0.37	444.4 ± 0.141	0.49	126.46 ± 0.836	97.1 ± 0.834	93.37 ± 0.220
LS2	3.40 ± 0.30	356.4 ± 0.250	0.57	127.23 ± 0.86	98.6 ± 0.374	97.09 ± 0.508
LS3	2.87 ± 0.07	387.5 ± 0.244	0.43	122.08 ± 0.675	95.6 ± 0.265	91.63 ± 0.321
LS4	3.21 ± 0.24	301.7 ± 0.068	0.55	117.05 ± 0.511	97.2 ± 0.604	94.87 ±0.131
LS5	3.63 ± 0.37	330.5 ± 0.128	0.47	127.34 ± 0.449	92.9 ± 0.547	93.45 ± 0.326
LS6	2.67 ± 0.16	486.5 ± 0.163	0.68	140.56 ± 0.311	91.9 ± 0.655	92.49 ± 0.399
LS7	2.94 ± 0.06	331.4 ± 0.331	0.51	118.67 ± 0.121	92.3 ± 0.753	96.53 ± 0.432
LS8	2.83 ± 0.01	446.5 ± 0.208	0.64	130.55 ± 0.362	94.4 ± 0.668	89.33 ± 0.218
LS9	3.56 ± 0.57	536.1 ± 0.264	0.54	138.02 ± 0.213	93.6 ± 0.351	90.09 ± 0.336
LS10	3.41 ± 0.37	341.7 ± 0.181	0.41	127.82 ± 0.173	99.7 ± 0.515	92.91 ± 0.183
LS11	4.34 ± 0.10	491.3 ± 0.331	0.60	136.44 ± 0.322	92.3 ± 0.532	95.47 ± 0.251

**Table 5:** Regression coefficient and ANOVA of independent factors.

Source	Disintegration Time (DT)	Angle of Repose of Powder	In Vitro Drug Release in 30 min
β°	11.28	5.35	9.66
A	0.2230	0.1469	0.0700
B	0.2990	0.1956	−0.1246
AB	−0.0077	−0.0060	0.0131
A^2^	0.0438	0.1882	0.0078
B^2^	0.0218	0.1736	0.0012
*p*-value	<0.0001	<0.0001	<0.0001
F-value	564.15	80.49	201.91
R^2^	0.9982	0.9877	0.9951
Adjusted.R^2^	0.9965	0.9755	0.9901
Predicted. R^2^	0.9956	0.9721	0.9784
Lack of Fit	0.9901	0.9997	0.7082

**Table 6:** Predicted and experimental values for three responses.

Responses	Optimum Ratio of Excipients (R)	Optimum Ratio of Drug Conc. (% cd)	Predicted Value	Experimental Value
Disintegration time	24.18%	15 mg	118.08	118.20 ± 0.148
Angle of repose	24.18%	15 mg	28.12	28.14 ± 0.562
Dissolution	25.22%	15 mg	95.61	95.75 ± 0.322

## Data Availability

Not applicable.
